# Landslide inventory dataset post Hurricane Stan (2005), Lake Atitlan region, Guatemala

**DOI:** 10.1016/j.dib.2022.108356

**Published:** 2022-06-07

**Authors:** Elia Axinia Machado, Yuri Gorokhovich, Mahta Ghahremani

**Affiliations:** a Department of Earth, Environmental, and Geospatial Sciences, Lehman College^1^, CUNY, Bronx, NY, USA; b Earth and Environmental Sciences, CUNY Graduate Center, New York, NY, USA; c Reilly Associates, Pittston, PA, USA

**Keywords:** Landslides, Hurricane impact, Hurricane Stan, Lake Atitlan, Natural hazards, Guatemala

## Abstract

Landslides are a global hazard of devastating impacts resulting in thousands of fatalities every year, substantial economic losses, and long-term economic disruption. Examining the frequency and distribution of landslides is important to better understand their associated factors and to identify where future landslides may occur. The dataset described in this article consists of landslides digitized as polygons using high resolution aerial photography collected after Hurricane Stan (October 2005), which caused severe damages in Central America. In Guatemala, the total economic impact of Hurricane Stan has been estimated at US$983 Million dollars, with 719,000 ha of land lost due to flooding, landslides, and erosion. The dataset is provided in shapefile format and encompasses the areas located north and northwest of Lake Atitlan. The digitizing process involved the visual identification of the landslides in the areal imagery followed by manual delineation of the landslides’ areal extent using ArcGIS desktop on-screen digitizing and editing tools. Additionally, a randomized verification procedure was completed to assess the completeness of the dataset.

## Specifications Table

**Table:** 

Subject	Earth and Planetary Sciences (Earth Surface Processes)
Specific subject area	Natural hazards (Landslides)
Type of data	Geographic Information Systems, shapefile (ESRI, Inc), polygon data. Python script.
How the data were acquired	Digitizing and editing tools in ArcMap 10.5 GIS software.
Data format	Raw, Analysed.
Description of data collection	Only the landslides that were visible from the orthophotos were digitized.
Data source location	The data were collected in two areas near Lake Atitlan, Guatemala. These include the Tzojoma area, northwest of the lake, and the Lake Atitlan area, north of Lake Atitlan (Fig. 1).
Data accessibility	The data can be downloaded from the OSF repository, doi 10.17605/OSF.IO/A8KP3
Related research article	Gorokhovich et al. [1] Improving landslide hazard and risk mapping in Guatemala using terrain aspect, Nat. Hazards, 81 (2016), 869–886. .

**Value of the Data**

• This data is useful for landslide modeling, as well as for assessing and mapping risk, vulnerability, and damages associated with landslides.
• Geomorphologists, geographers, insurance companies, and geologists interested in natural hazards can benefit from this data.
• This data can be used as input in landslide susceptibility models, to help predict landslide occurrences after hurricanes, and to inform land use planning processes that incorporate hazard management and prevention.

## 1 Data Description

We use the term landslide to refer to a “a rapid displacement of a mass of rock, residual soil, or sediments adjoining a slope, in which the center of gravity of the moving mass advances in a downward and outward direction” [2] as well as the landform resulting from such movement [3]. Examining the frequency and distribution of landslides is important to better understand their associated factors, but also to identify indicators of future landslide risk.

**Fig. 1:**
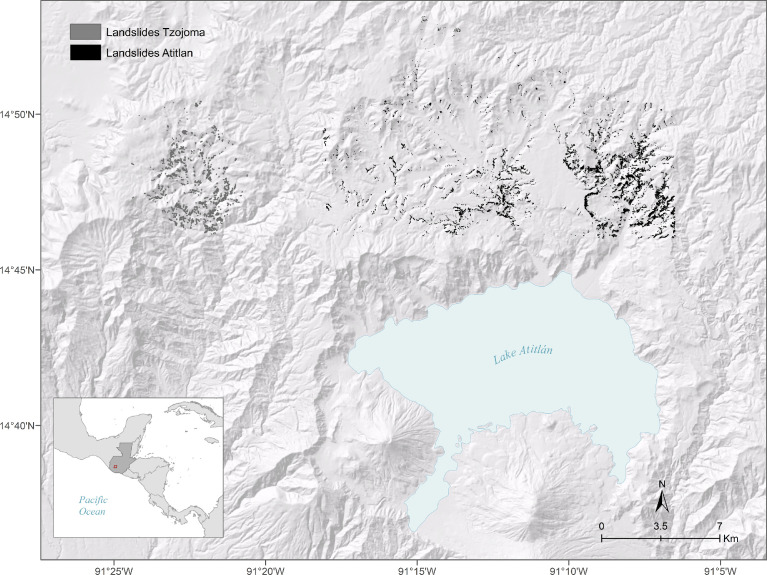
Overview of the digitized landsides included in the dataset overlaid on a hillshaded digital elevation model.

The digitized landslides included in this dataset are debris flows for the most part, which can be triggered by intense rainfall periods [3], such as those associated with Hurricane Stan. Fig. 1 shows an overview of the digitized landslides in the areas of Lake Atitlan and Tzojoma.

**Fig. 2:**
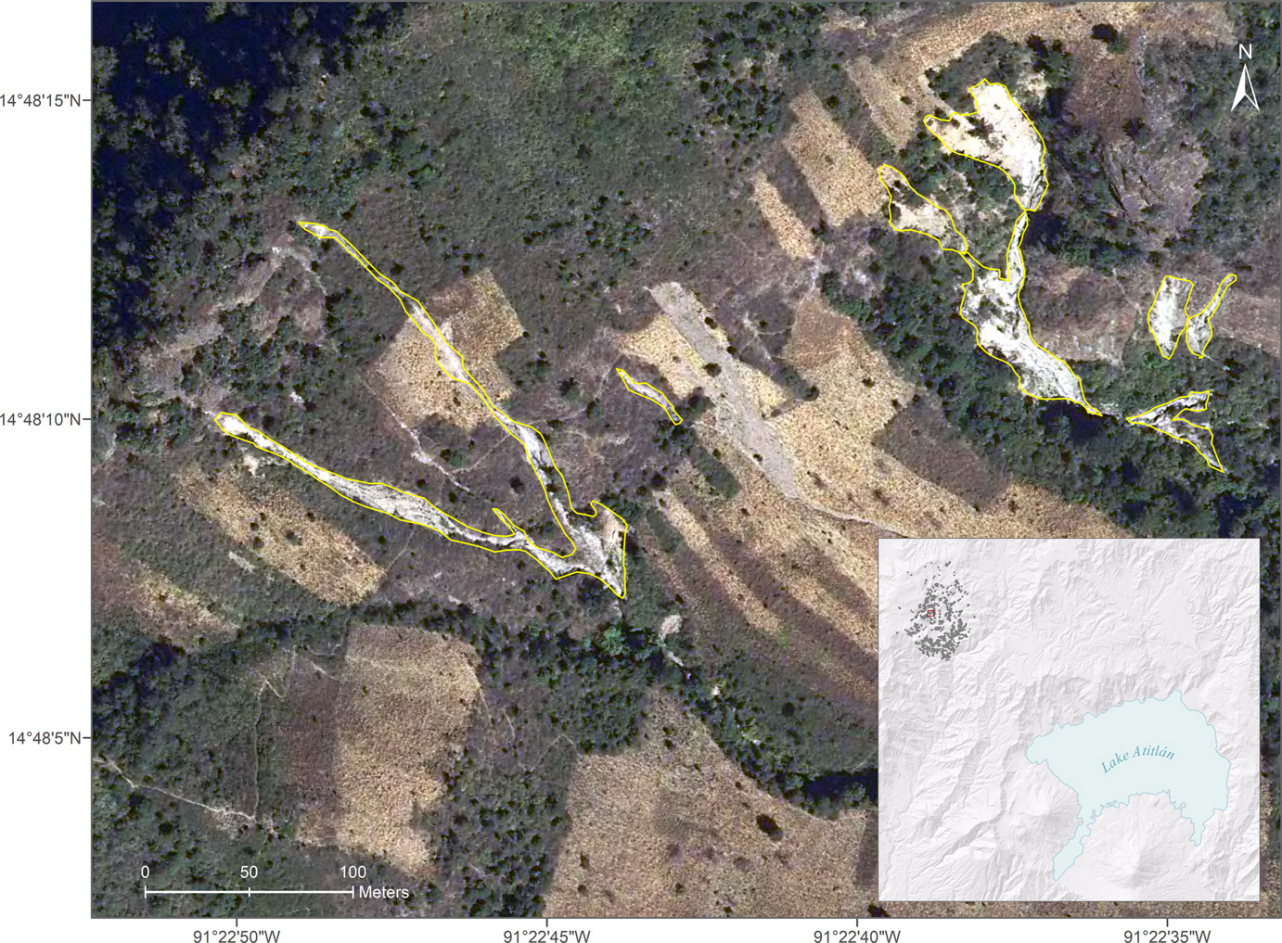
Subset of digitized landslides in the Tzojoma area.

**Fig. 3:**
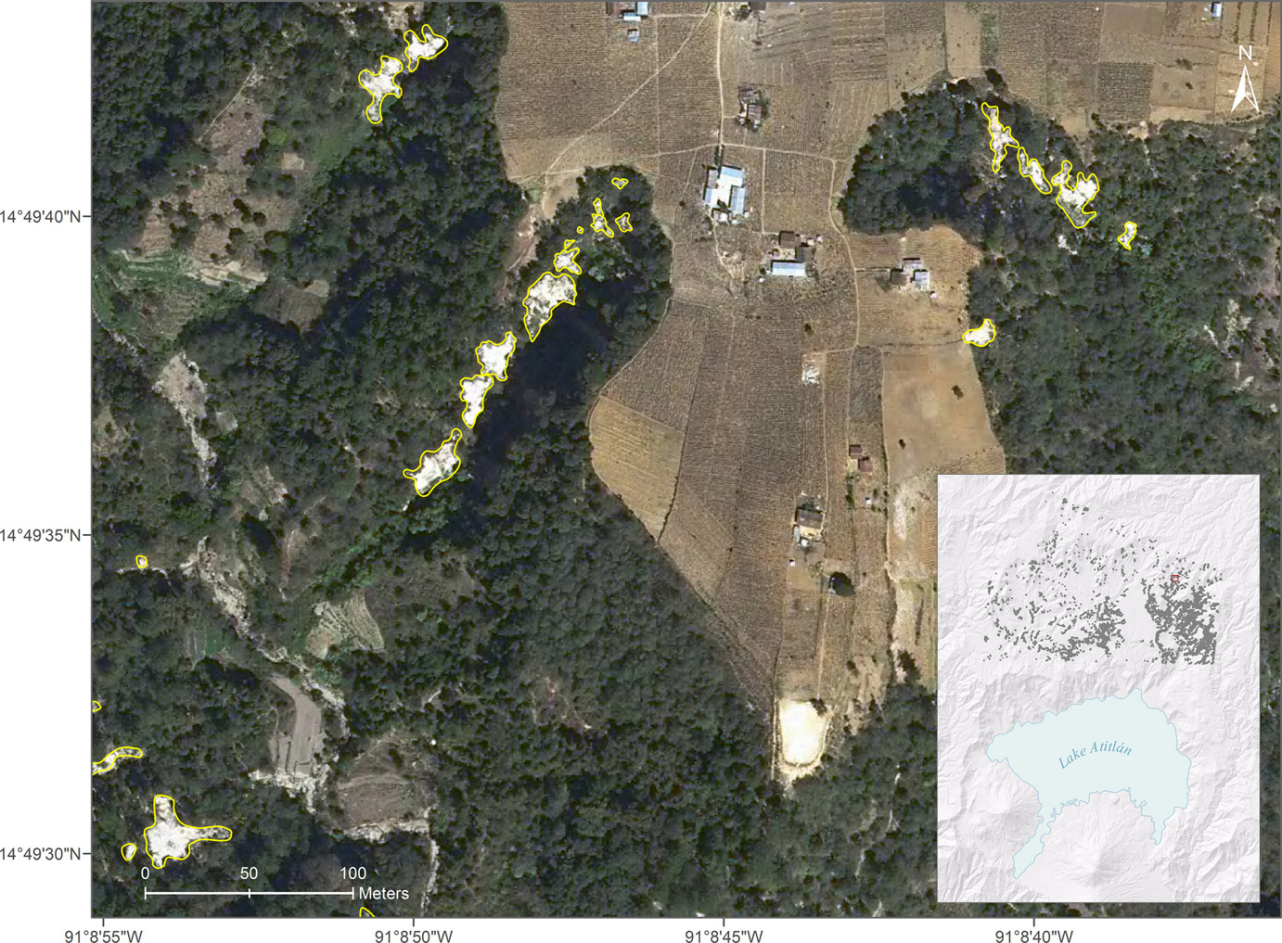
Subset of digitized landslides in the Lake Atitlan area.

Most of the landslides are concentrated in areas of medium to steep hillslopes in proximity to roads, and riverbeds, and to agricultural and populated areas to a lesser extent (Figs. 2, 3).

The area of the landslide polygons ranges from 3.60 m^2^ to 1.32 ha in the Lake Atitlan area and from 4.35 m^2^ to 0.94 ha the Tzojoma area (Table 1).

**Table 1:** Descriptive statistics of digitized landslides (areas in square meters).

	Landslides (count)	Minimum Area	Maximum Area	Average Area	Std. Deviation (area)	Total Area
**Atitlan**	6817	3.60	13,223.22	358.32	716.91	2,442,638.92
**Tzojoma**	962	4.35	9410.45	520.89	925.30	501,100.48

The dataset consists of six shapefiles:

• Landslides_Atitlan_UTM15N_6817: Includes 6817 polygons of digitized landslides in the Lake Atitlan area.
• Landslides_Tzojoma_UTM15N_962: Includes 962 polygons of digitized landslides in the Tzojoma area.
• Boundary_Tzojoma_UTM15N: Polygon boundary of the Tzojoma area used to digitize the landslides.
• Boundary_Atitlan_UTM15N: Polygon boundary of the Lake Atitlan area used to digitize the landslides.
• Fishnet_Atitlan_UTM15N_932: Fishnet polygon for the Lake Atitlan area with the completeness results.
• Fishnet_Tzojoma_UTM15N_217: Fishnet polygon for the Tzojoma area with the completeness results.

The shapefiles of the digitized landslides include a features ID (FID) and an area field (AreaSqm) in their attribute tables specifying the area of each landslide polygon in square meters. The coordinate system for all shapefiles is UTM 15 N (WGS84). The fishnet shapefiles include the number of total landslides per grid selected (TotLand), the total number of landslides that were digitized (TotDig), the percent completeness value (ComPc, a value of 999 indicates that the percent completeness was not calculated because the grid was not selected for the completeness evaluation or its total number of landslides was zero), and AreaSqm (the area of the grid in square meters). Additionally, we also provide the polygons defining the bounding extent of the digitized landslides in each region including its area in square kilometres (AreaSqKm).

## 2 Experimental Design, Materials and Methods

Landslide inventories and maps have been provided as points, e.g., [4], polygons, e.g., [1,^5^,6], or density grids, e.g., [7]. We chose to digitize the landslides as polygons to provide more flexibility for landslide susceptibility and damage analysis, since this method allows to estimate the area, volume, and run-up distance of landslides, which is not possible to obtain from points. In addition, digitizing the landslides as polygons allows to represent the heterogeneity of the topographic and geologic factors associated with the landslide occurrences (e.g., slope, aspect, slope curvature, geology, and soil composition). This heterogeneity could be substantial depending on the size of the landslide and the spatial resolution of the data used to assist the digitizing process.

The landslides were digitized manually as polygons with the editing tools in ArcMap 10.5 GIS software following an on-screen digitizing process. To aid in the identification of the landslides, we used a point layer of the landslides associated with Hurricane Stan in the Tzojoma area as well as high resolution orthorectified aerial imagery (0.5 m spatial resolution) provided by the NGO Vivamos Mejor in Guatemala.. Additionally, we generated a fishnet polygon shapefile (500 m grid size) covering the extent of both areas of interest to guide the digitizing process.

The orthorectified aerial imagery covers the country of Guatemala and was captured between December 2005 and March 2006. It was sourced by the Guatemalan Ministry of Agriculture, Livestock and Food (Ministerio de Agricultura Ganadería y Alimentación, MAGA) and is accessible through a Web Map Services (WMS)^2^ and the geoportal of the Guatemalan Secretariat for Planning and Programming of the Presidency (Secretaría de Planificación y Programaión de la Presidencia (SEGEPLAN) (http://ideg.segeplan.gob.gt/geoportal/).

The digitizing process was performed for each area of interest separately and involved overlaying the fishnet polygon over the orthorectified areal imagery and the point layer (Tzojoma area only). Subsequently, and following [8] we zoomed to the first grid polygon in the fishnet at scale 1:1000 and started an iterative process of manually digitizing the areal extent of visible landslides in the top left grid, panning to the next grid until all the grids with visible landslides were digitized. A zoom level corresponding to a scale of 1:1000 was maintained throughout the digitizing process for spatial consistency.

### 2.1 Completeness

We aimed to constrain the digitizing to the landslides that contrasted the most with the background (i.e., had a stronger white color) since those are more likely to be associated with Hurricane Stan. However, landslides can be confused with eroded terrain, particularly near rivers and roads, as well as exposed rocks, resulting in commission errors (i.e., false positives). Additionally, and despite our best efforts to exclude older (i.e., darker) landslides, it is very possible that the dataset also includes older landslides that may have occurred after Hurricane Stan since the exact date of the landslides ocurrences is not certain. Likewise, it is also possible that some landslides were missed (omission), which can occur in areas with shadows associated with hillslopes or forest cover, for example. We assessed this issue using a conservative approach to evaluate the completeness of the dataset described below.

The provided dataset is a revised subset of the data used in the paper Gorokhovich et al. [1] and includes a revision process for completeness. This process involved using the polygon fishnet mentioned above to evaluate the percent completeness of grids selected at random. First, we generated random numbers using a python script to select approximately 50% of the grids based on their FID in the Tzojoma region and 20% of the grids in the Lake Atitlan region. Specifically, 109 grids (out of 217) were selected in the Tzojoma region (covering 51.72% of the grid area) and 187 (out of 932) in the Lake Atitlan region (covering 19.94% of the grid area).^3^ Second, we panned to each randomly selected grid noting the total potential number of landslides as well as the total number of landslides digitized to calculate the percent completeness for each grid as follows:

(Total digitized landslides/total potential landslides) * 100 Eq.(1).

It is important to note that this completeness assessment is very conservative since the criteria for landslide inclusion in the evaluation, particularly with regards to its color, was less strict than during the digitizing process. As a result, a landslide that was not digitized initially was counted as a potential landslide during the completeness assessment when in doubt it could have been associated with Hurricane Stan. Also, the completeness is calculated for grids that contain at least one landslide, excluding any grids that do not contain any landslides that were correctly considered as such during the digitalization process.

Out the of 109 grids randomly selected in the Tzojoma area, 67 contained at least one landslide, with an average completeness of 88.49%. In the Lake Atitlan area, 87 grids, out of the 187 selected contained at least one landslide and had an average completeness percent of 93.59. A copy of the python script used to generate the random numbers for the completeness assessment (Random_NumGrd.py), which outputs two CSV files with the random numbers for each study area is also provided.

### 2.2 Dataset Usage Considerations

The different parts of the landslides, such as the main scarp, have not been digitized separately, which may be of special interest for some analysis. Additionally, it was not possible to exclude vegetation from the boundary of the landslides in most cases, therefore the landslide polygons include vegetation. The polygons may also include exposed rocks or soil that may not be associated with the landslide event. Lastly, the spatial resolution of the orthorectified areal imagery and digitizing scale limits the digitized landslides to those measuring 1 m wide or more.

## CRediT authorship contribution statement

**Elia Axinia Machado:** Conceptualization, Methodology, Validation, Formal analysis, Software, Resources, Data curation, Writing – original draft, Writing – review & editing, Visualization, Supervision, Funding acquisition. **Yuri Gorokhovich:** Conceptualization, Methodology, Resources, Writing – original draft, Supervision. **Mahta Ghahremani:** Data curation.

## Declaration of Competing Interest

The authors declare that they have no known competing financial interests or personal relationships that could have appeared to influence the work reported in this paper.

## Data Availability

Machado, E.A., Gorokhovich, Y., Gahremani, M. 2022. Landslide dataset post Hurricane Stan (2005), Lake Atitlan region, Guatemala (Original data) (OSF repository) doi 10.17605/OSF.IO/A8KP3.

## Footnotes

2 Geographic Information System (GIS) software is required to visualize the orthophotos using this link: http://ide.segeplan.gob.gt/cgi-bin/mapserv.exe?map=/ot_web/ot_overview_1.map&SERVICE=WMS& To access the orthophotos using ArcGIS desktop: 1. Open ArcMap and double click on “add WMS Server” in the ArcCatalog. 2. Paste the WMS link provided above in the URL field in the new window that appears and click OK. 3. The orthophotos service "Orthophotos on ide.segeplan.gob.gt" is added to ArcCatalog. 4. Click the plus sign next to it to expand its contents. 5. Drag the layers including “landsat_img” (false color Landsat composite of Guatemala) to the table of contents for visualization.

3 Some grids were not exactly 250,000 sqm because they were at the borders of the landslide digitizing extent and were partially cut off.
